# Efficacy of Cytokine-Induced Killer Cell Immunotherapy for Patients With Pathologically Pure Glioblastoma

**DOI:** 10.3389/fonc.2022.851628

**Published:** 2022-04-08

**Authors:** Myung-Hoon Han, Jae Min Kim, Jin Hwan Cheong, Je Il Ryu, Yu Deok Won, Gun He Nam, Choong Hyun Kim

**Affiliations:** ^1^Department of Neurosurgery, Hanyang University Guri Hospital, Guri, South Korea; ^2^Development Division, GC Cell Corp., Yongin-si, South Korea

**Keywords:** glioblastoma multiforme, cytokine-induced killer cell (CIK), immunotherapy, adoptive cell transfer, overall survival

## Abstract

The most common malignant central nervous system tumor is glioblastoma multiforme (GBM). Cytokine-induced killer (CIK) cell therapy is a promising type of adoptive cell immunotherapy for various cancers. We previously conducted a randomized clinical trial on CIK cell therapy in patients with GBM. The aim of this study was to evaluate the efficacy of CIK immunotherapy for patients with pathologically pure GBM, using data from our previous randomized clinical trial. The difference between overall survival (OS) and progression-free survival (PFS) according to CIK immunotherapy was analyzed using the Kaplan–Meier method. Hazard ratios were calculated using univariate and multivariate Cox regression analyses to determine whether CIK cell immunotherapy was independently associated with higher OS and PFS in patients with pure GBM. A total of 156 eligible patients were included in the modified intention-to-treat (mITT) population. We confirmed that 125 (80.1%) GBM samples were pure GBM tumors without the presence of other types of tumors. For patients with pure GBM, Kaplan-Meier analysis showed no significant difference in OS between the CIK cell treatment and control groups. However, multivariate Cox regression demonstrated CIK cell immunotherapy as an independent predictor of greater OS (hazard ratio, 0.59; 95% CI, 0.36–0.97; p = 0.038) and PFS (hazard ratio, 0.55; 95% CI, 0.36–0.84; p = 0.001) in patients with pathologically pure GBM in the mITT population. This study showed that CIK cell immunotherapy combined with conventional temozolomide chemoradiotherapy could prolong OS and PFS in patients with newly diagnosed pathologically pure GBM, with no significant adverse events related to treatment. However, unlike the results of multivariate Cox analysis, no statistical significance of CIK cell immunotherapy in OS in Kaplan-Meier analysis raises a question. Further studies are required to validate these results.

## 1 Introduction

The most common malignant central nervous system (CNS) tumor is glioblastoma multiforme (GBM) (48.3%), which accounts for approximately 41.8–57.3% of gliomas (1, 2). The standard treatment for glioblastoma consists of surgical resection and standard temozolomide (TMZ) chemoradiotherapy (3). Despite these treatments, the median survival of patients with glioblastoma is only 14.6 months (3).

Recently, success with using adoptive immunotherapy and checkpoint inhibitors for various types of cancers has attracted interest in immune-targeted strategies for the treatment of GBM (4–7). However, the CNS is thought of as an immune-privileged site with the restricted access of immune cells to the brain due to the blood-brain barrier (BBB) (8). Nevertheless, the concept of the immune privilege of the CNS has been redefined because studies have reported that activated T cells can cross the BBB and diffusely penetrate the brain parenchyma (9–13). Adoptive immunotherapy is a highly personalized cancer therapy and one of the most promising immunotherapies, and its efficacy and safety have been proven in various cancers (14). Cytokine-induced killer (CIK) cells are major histocompatibility (MHC)-unrestricted cytotoxic natural killer (NK)-like T cells that can be generated from peripheral blood lymphocytes by *ex vivo* incubation with the addition of interferon (IFN)-γ, interleukin (IL)-2, and CD3 monoclonal antibody (15, 16). CIK cell therapy is a promising type of adoptive cell immunotherapy, and several clinical trials involving CIK cells have been conducted for various cancers (17).

In 2017, although bevacizumab (Avastin) did not significantly improve overall survival (OS) in patients with GBM, the U.S. Food and Drug Administration (FDA) granted full approval of Avastin for the treatment of recurrent glioblastoma. In a previous randomized clinical trial, we also showed that CIK cell immunotherapy combined with standard TMZ chemoradiotherapy prolonged PFS but not OS in the GBM group compared with the control group (18). Therefore, through subgroup analysis, we aimed to further investigate whether CIK cell therapy could affect OS in GBM patients.

We only examined patients with pathologically pure GBM considering that GBM is known to be occasionally present with other types of tumors (19–22). We hypothesized that the efficacy of adoptive CIK cell immunotherapy may be considerably different between the treatment and control groups. This additional study was possible because we initially performed an independent pathology review of all patients with GBM prior to randomization (18). Therefore, pathological findings were available for all the patients. Phase III trials are well known as the best way to find a new standard for treatment, as this process takes much effort and time. Therefore, depending on the results of this trial phase, FDA approval may be obtained or the patient’s treatment may be changed. Since GBM is the worst malignant tumor in the CNS, and it induces an immunosuppressive tumor microenvironment, there are high expectations for the efficacy of immunotherapy in the treatment of GBM. Therefore, extensive studies related to immunotherapy for GBM have been conducted, and many clinical trials are under way (23). Randomized clinical trials of immunotherapy in GBM are important for medical advances related to CNS malignant brain tumors. Therefore, there was need for further evaluation through subgroup analysis in our previous Phase III, multi-center clinical trial. In this study, we aimed to evaluate the efficacy of CIK cell immunotherapy in addition to conventional TMZ chemoradiotherapy for patients with pathologically pure GBM using the data of our previous randomized clinical trial.

## 2 Materials and Methods

### 2.1 Study Design and Participants

This study was originally performed on patients with newly diagnosed GBM as a randomized, open-label, phase III multi-center trial from December 2008 to October 2012 (clinicaltrials.gov NCT 00807027) (18). The trial was performed at 7 Korean university hospitals, and all participants were registered before the start of concomitant TMZ with radiation therapy (RT). The eligibility criteria in this study were: (1) 18 to 70 years of age; (2) a Karnofsky Performance Status (KPS) of at least 60; (3) newly diagnosed GBM as confirmed on central review with adequate hematologic, renal, and hepatic function. Patients were excluded if they had immune-related diseases and other conditions as previously described (18). Patients who met the eligibility criteria were randomly assigned (1:1) to receive either autologous CIK cell immunotherapy combined with standard chemoradiotherapy with TMZ or standard TMZ chemoradiotherapy alone. Random assignment *via* an Interactive Voice Response System (IVRS), masking procedures, and real-time monitoring of safety events were performed as previously described (18). The data were collected by the sponsor, who vouched for data accuracy, and subgroup analysis was performed by the investigator.

The study protocol was approved by the local institutional review boards (IRB No. KUH1070007; KMC IRB 0849-01; AN08087; 4-2008-0387; 2008-07-058; 2008-0320) prior to patient enrollment and adhered to the tenets of the Declaration of Helsinki. This trial was also approved by the Ministry of Food and Drug Safety of Korea.

### 2.2 Pathology Review

After obtaining informed consent and prior to randomization, we additionally performed an independent pathology review of all tumor tissues (18). As GBM is occasionally present with anaplastic astrocytoma, oligodendroglioma, PNET, or sarcoma (19–22, 24), we sought to determine whether there is a difference in prognosis after adjuvant CIK immunotherapy between pure and mixed glioblastomas. Therefore, we newly classified the patients into the pure GBM group, GBM with astrocytoma (anaplastic or gemistocytic) group, GBM with oligodendroglial tumor group, and GBM with others (PNET or sarcomatous change) group in this study.

### 2.3 Standard Chemoradiation Treatment Protocol

Both the CIK immunotherapy and control groups received standard TMZ chemoradiotherapy (3). Concurrent radiotherapy (60 Gy in 30 fractions) and TMZ (75 mg/m^2^ per day) were initially delivered for 6 weeks after surgery. After 4 weeks, the patients received six maintenance cycles of adjuvant TMZ (150–200 mg/m^2^/day for the first 5 days of a 28-day cycle) if treatment-related adverse events had not occurred.

### 2.4 Production of CIK Cells and Adoptive Immunotherapy Protocol

For the treatment of autologous adoptive CIK immunotherapy, peripheral blood (> 120 ml) was obtained from each patient in the CIK immunotherapy group at least 2 weeks before CIK cell agent administration. CIK cells were generated at a GMP-certified central facility (GC CELL Corp., Yongin, Korea) as previously described (25). CIK cells were activated using immobilized anti-CD3 antibody (Orthoclone OKT3; Janssen, Beerse, Belgium) and recombinant interleukin-2 (Proleukin; Novartis, Basel, Switzerland). Peripheral mononuclear cells (PBMCs) obtained from the patients were isolated by Ficoll density gradient centrifugation. The separated cells were suspended at a concentration of 0.3–3 × 10^6^ cells/ml in media (GC Lymphotec, Tokyo, Japan), and they were cultured for 5 days in a flask coated with human anti-CD3 antibody. Subsequently, they were cultured in media containing recombinant human IL-2 for 14 days and used in the experiment. After culture for 14 days, the total number of cells was increased by around 200 times.

**Figure 1:**
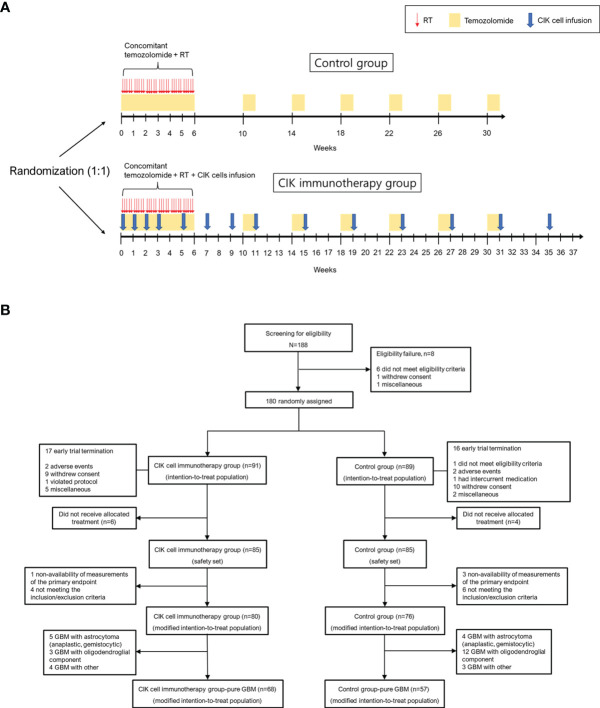
Overall study protocol. **(A)** Schematic overview of the trial. **(B)** Patient disposition. CIK, cytokine-induced killer.

Patients in the CIK immunotherapy group received the CIK cell agent intravenously over 60 min and were observed for at least 30 min at an outpatient clinic. The CIK cell agent contained an average of 6.55 × 10^9^ cells per treatment as previously described (18). The patients were scheduled to receive the CIK cell agent a total of 14 times (4 times once a week, followed by 4 times every 2 weeks and 6 times every 4 weeks) in addition to the standard TMZ chemoradiotherapy **(** **Figure 1A****)**.

### 2.5 Endpoints and Assessment of Clinical Responses

The primary endpoints were OS and progression-free survival (PFS), and the secondary endpoint was safety. OS was measured from the date of randomization until death from any cause. PFS was defined as the time interval between the date of randomization and first evidence of tumor progression or death (26). PFS was assessed based on enhanced MRI performed approximately 4 weeks after chemoradiotherapy, 10, 22, 34, and 46 weeks after randomization, and every 3~12 months thereafter during the follow-up period. In addition to the investigators who assessed tumor progression, two radiologists at an independent review facility reviewed all of the MRI scans. The independent reviewers were blinded to the study-group assignments, and were granted read-only access to previous reviews until the final imaging dataset was analyzed (18). Adverse events were classified and graded according to the National Cancer Institute Common Terminology Criteria for Adverse Events (CTCAE), version 3.0.

### 2.6 TCGA Database

We additionally obtained the TCGA dataset to compare the OS and PFS between the CIK cell immunotherapy group and control group (larger cohort of patients with GBM who also received standard TMZ chemoradiotherapy). For the TCGA dataset, the clinical information of 619 GBM patients was initially downloaded from the TCGA database (https://gdc.cancer.gov/about-data/publications/pancanatlas and https://www.cbioportal.org/). Patients without information on chemoradiotherapy or those who received immunotherapy were excluded from the 619 patients with GBM in the TCGA database.

### 2.7 Statistical Analysis

The intention-to treat (ITT) population was defined as all participants who were randomized in the clinical study **(** **Figure 1B****)**. The modified ITT (mITT) population was defined as follows: (1) participants who received at least one allocated treatment during the study period; (2) participants who received at least one efficacy assessment during the study period; (3) participants who met the inclusion and exclusion criteria during the study period. The safety population was defined as all participants who received at least one allocated treatment during the study period for safety assessment.

The chi-square and Student’s *t*-test were performed to evaluate differences between the CIK immunotherapy and control groups. Statistical analysis was conducted with a focus on the pure GBM group.

The difference between OS and PFS according to CIK immunotherapy was analyzed using the Kaplan–Meier method with log-rank test. Hazard ratios (HRs) with 95% confidence intervals (CIs) were then calculated with uni- and multivariate Cox regression analyses to determine whether CIK immunotherapy is independently associated with higher OS and PFS in patients with pure GBM.

A p value < 0.05 was considered statistically significant. All statistical analyses were performed using R software version 3.6.3 and SPSS for Windows version 24.0 (IBM, Chicago, IL).

## 3 Results

### 3.1 Patient Characteristics

Between December 2008 and October 2012, 188 patients from 7 institutes in Korea were screened. A total of 180 eligible patients were assigned randomly to either the CIK cell immunotherapy group (91 patients) or the control group (89 patients) (ITT population). The mITT population comprised 156 patients, and the number of patients with pure GBM in the mITT population was 125 (68 and 57 assigned to the CIK cell immunotherapy and control groups, respectively) **(** **Figure 1B****)**. The mean age at the time of randomization was 53.3 years, and 43.6% of patients were women in the mITT population **(** **Table 1****)**. Based on an independent pathology review, we confirmed that 125 (80.1%) GBM samples were pathologically pure GBMs without the presence of any other gliomas or other types of tumors in the mITT population. There were no significant differences in characteristics between the two treatment groups. The detailed information of the patients is shown in **Table 1**. Although not statistically significant, patients with pathologically mixed GBM showed higher OS and PFS than patients with pathologically pure GBM in the mITT population **(** **Supplementary Figure 1****)**.

**Table 1:** Clinical characteristics of patients with GBM in the modified ITT population.

Characteristics	Control group (n = 76)	CIK immunotherapy group(n = 80)	Total(n = 156)	p
Sex, female, n (%)	33 (43.4)	35 (43.8)	68 (43.6)	0.967
Age, mean ± SD, y	53.3 ± 10.2	53.2 ± 10.7	53.3 ± 10.4	0.956
Time duration between randomization and death (months), mean ± SD	17.8 ± 10.2	18.8 ± 10.7	18.4 ± 10.5	0.554
Time duration between randomization and disease progression (months), mean ± SD	8.6 ± 8.4	11.1 ± 9.8	9.9 ± 9.2	0.081
Pathology review, n (%)				0.089
Pure GBM	57 (75.0)	68 (85.0)	125 (80.1)	
GBM with astrocytoma (anaplastic or gemistocytic)	4 (5.3)	5 (6.3)	9 (5.8)	
GBM with oligodendroglial tumor	12 (15.8)	3 (3.8)	15 (9.6)	
GBM with others	3 (3.9)	4 (5.0)	7 (4.5)	
Karnofsky performance scale score, median (IQR)	90 (80–100)	90 (80–100)	90 (80–100)	0.439
Extent of resection, n (%)				0.708
Biopsy only	6 (7.9)	10 (12.5)	16 (10.3)	
Partial resection	5 (6.6)	6 (7.5)	11 (7.1)	
Subtotal resection	19 (25.0)	22 (27.5)	41 (26.3)	
Gross total resection	46 (60.5)	42 (52.5)	88 (56.4)	
Steroid use, n (%)				0.350
No	22 (28.9)	25 (31.1)	47 (30.1)	
Before allocated treatment	3 (3.9)	1 (1.3)	4 (2.6)	
During allocated treatment	28 (36.8)	22 (27.5)	50 (32.1)	
Both before and during allocated treatment	23 (30.3)	32 (40.0)	55 (35.3)	

ITT, intention-to-treat; GBM, glioblastoma multiforme; CIK, cytokine-induced killer; SD, standard deviation; IQR, interquartile range.

### 3.2 Efficacy of CIK Immunotherapy

#### 3.2.1 Overall Survival

**Figure 2:**
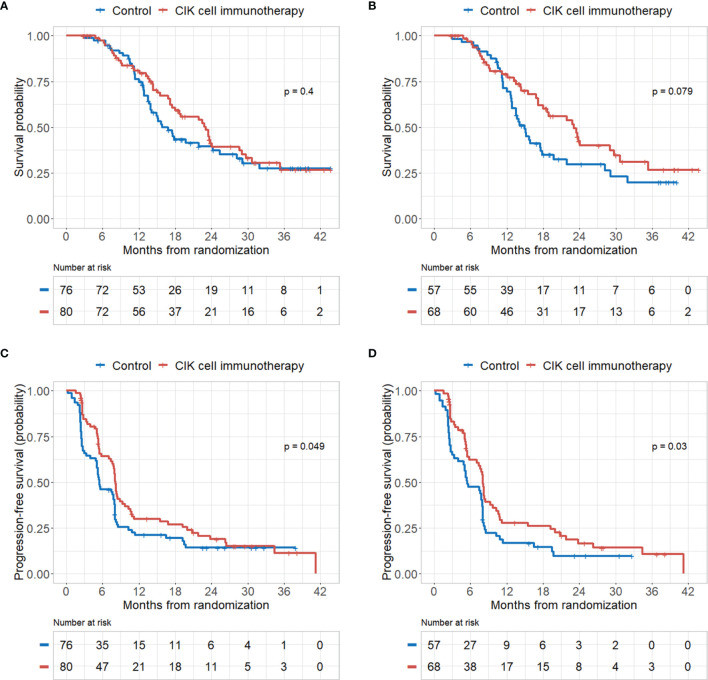
Kaplan–Meier curves of overall survival (OS) and progression-free survival (PFS) rates according to CIK immunotherapy in the mITT population and mITT population with pathologically pure GBM. **(A)** OS rate according to CIK immunotherapy for patients in the mITT population. **(B)** OS rate according to CIK immunotherapy for patients with pure GBM in the mITT population. **(C)** PFS rate according to CIK immunotherapy for patients in the mITT population. **(D)** PFS rate according to CIK immunotherapy for patients with pure GBM in the mITT population.

There were no significant differences in OS rates between the CIK cell immunotherapy and control groups in the mITT population **(** **Figure 2A****)**. When only pure GBM patients were analyzed in the mITT population, the difference in OS was more prominent between the CIK immunotherapy and control groups; however, it was not statistically significant **(** **Figure 2B****)**. The median OS rates of the CIK immunotherapy and control groups were 23.1 months and 14.9 months, respectively, for pure GBM patients in the mITT population **(** **Figure 2B****)**. In the multivariate Cox regression analysis, CIK cell immunotherapy was an independent predictor of greater OS for pure GBM patients in the mITT population (HR, 0.59; 95% CI, 0.36–0.97; p = 0.038) **(** **Table 2****)**. We found that the statistical significance of differences in OS and PFS between the CIK cell treatment and control groups was increased when the extent of resection variable was adjusted in the multivariate analysis among patients with pure GBM in the mITT population. Although not statistically significant, we observed that the rate of biopsy was higher and the rate of gross total resection was lower in the CIK cell immunotherapy group than in the control group in patients with pure GBM in the mITT population (gross total resection, 50.0% vs. 63.2%; biopsy only, 11.8% vs. 5.3%) **(** **Table 3****)**.

**Table 2:** Overall survival and progression-free survival analyses according to the clinical parameters of patients with pure GBM in the modified ITT population.

Variable	Overall survival				Progression-free survival			
	Univariate analysis		Multivariate analysis		Univariate analysis		Multivariate analysis	
	HR (95% CI)	p	HR (95% CI)	p	HR (95% CI)	p	HR (95% CI)	p
Sex								
Male	Reference		Reference		Reference		Reference	
Female	0.73 (0.46–1.17)	0.191	0.63 (0.39–1.01)	0.056	0.84 (0.56–1.25)	0.383	0.81 (0.54–1.22)	0.318
Age (per 1 year increase)	1.00 (0.98–1.02)	0.957	1.00 (0.97–1.02)	0.740	1.00 (0.98–1.01)	0.707	0.99 (0.97–1.01)	0.445
BMI (per 1 BMI increase)	1.05 (0.97–1.14)	0.203	1.03 (0.95–1.13)	0.455	1.08 (1.00–1.16)	**0.048**	1.10 (1.02–1.19)	**0.015**
Karnofsky performance scale score (per 10 score increase)	0.99 (0.97–1.01)	0.230	0.98 (0.96–1.00)	**0.042**	1.00 (0.98–1.01)	0.693	0.99 (0.97–1.01)	0.161
Extent of resection								
Biopsy only	Reference		Reference		Reference		Reference	
Partial resection	1.47 (0.53–4.06)	0.458	2.42 (0.81–7.25)	0.115	0.70 (0.28–1.72)	0.430	0.65 (0.25–1.69)	0.380
Subtotal resection	0.43 (0.17–1.10)	0.079	0.48 (0.18–1.28)	0.143	0.31 (0.14–0.67)	**0.003**	0.24 (0.11–0.54)	**0.001**
Gross total resection	0.52 (0.22–1.24)	0.142	0.56 (0.23–1.38)	0.211	0.36 (0.18–0.74)	**0.005**	0.23 (0.11–0.51)	**< 0.001**
Steroid use								
No	Reference		Reference		Reference		Reference	
Before allocated treatment	0.89 (0.12–6.77)	0.911	0.94 (0.12–7.33)	0.949	0.38 (0.05–2.84)	0.349	0.34 (0.05–2.54)	0.292
During allocated treatment	2.07 (1.12–3.83)	**0.021**	2.23 (1.17–4.23)	**0.015**	1.73 (1.03–2.90)	**0.037**	1.60 (0.94–2.74)	0.083
Both before and during allocated treatment	1.54 (0.82–2.88)	0.181	1.33 (0.68–2.63)	0.409	1.10 (0.66–1.83)	0.724	0.79 (0.45–1.38)	0.402
Treatment group								
Control group	Reference		Reference		Reference		Reference	
CIK immunotherapy group	0.67 (0.43–1.05)	0.081	0.59 (0.36–0.97)	**0.038**	0.65 (0.44–0.96)	**0.032**	0.55 (0.36–0.84)	**0.001**

ITT, intention-to-treat; GBM, glioblastoma multiforme; HR, hazard ratio; CI, confidence interval; BMI, body mass index; CIK, cytokine-induced killer; p < 0.05 is shown in bold.

**Table 3:** Comparison of extent of resection between the CIK cell immunotherapy group and control group in patients with pure GBM in the mITT population.

Characteristics	Control group (n = 57)	CIK cell immunotherapy group (n = 68)	Total (n = 125)	p
Extent of resection, n (%)				0.242
Biopsy only	3 (5.3)	8 (11.8)	11 (8.8)	
Subtotal or partial resection	18 (31.6)	26 (38.2)	44 (35.2)	
Gross total resection	36 (63.2)	34 (50.0)	70 (56.0)	

CIK, cytokine-induced killer; GBM, glioblastoma multiforme; mITT, modified intention-to-treat.

Differences in OS rates between the CIK cell immunotherapy and control groups in the ITT population according to the presence of pathologically pure GBM were determined **(** **Supplementary Figures 2A, B****)**. Similar to the mITT population, CIK cell immunotherapy was an independent predictor of higher OS for pure GBM patients in the ITT population (HR, 0.62; 95% CI, 0.39–0.97; p = 0.036) **(** **Supplementary Table 1****)**.

#### 3.2.2 Progression-Free Survival

PFS rates were significantly higher in the CIK cell immunotherapy group compared with the control group for both the mITT population and pure GBM patients in the mITT population (p = 0.049 and p = 0.030, respectively) **(** **Figures 2C, D****)**. The median PFS rates of the CIK cell immunotherapy and control groups were 8.1 months and 5.5 months, respectively, for pure GBM patients in the mITT population **(** **Figure 2D****)**. In multivariate Cox regression analysis, CIK immunotherapy was an independent predictor of greater PFS for pure GBM patients in the mITT population (HR, 0.55; 95% CI, 0.36–0.84; p = 0.001) **(** **Table 2****)**.

Similarly, we observed significantly higher PFS rates in the CIK cell immunotherapy group compared with the control group for both the ITT population and pure GBM patients in the ITT population **(** **Supplementary Figure 2C, D****)**. In addition, CIK cell immunotherapy was an independent predictor of higher PFS for pure GBM patients in the ITT population **(** **Supplementary Table 1****)**.

#### 3.2.3 Additional Validation of the Efficacy of CIK Immunotherapy in an Independent Cohort

**Figure 3:**
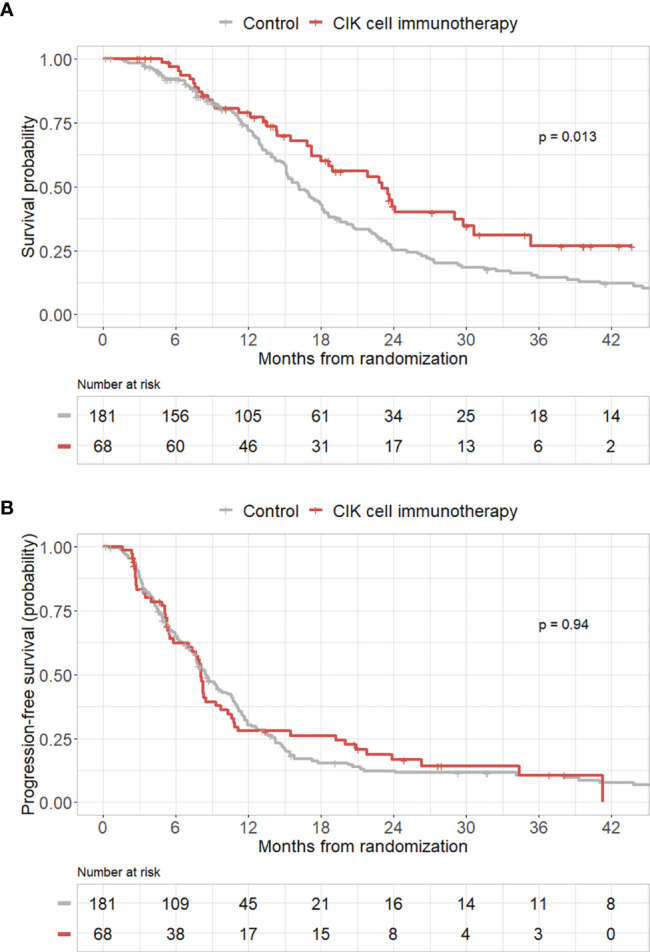
Kaplan–Meier curves of overall survival (OS) and progression-free survival (PFS) rates according to CIK immunotherapy in the mITT population with pathologically pure GBM with the TCGA cohort as the control group. **(A)** OS rate according to CIK immunotherapy for patients with pure GBM in the mITT population with the TCGA cohort as the control group. **(B)** PFS rate according to CIK immunotherapy for patients with pure GBM in the mITT population with the TCGA cohort as the control group.

To validate the efficacy of CIK immunotherapy in an independent cohort, we used the publicly accessible TCGA database. A total of 181 patients with GBM who received both surgery and standard TMZ chemoradiotherapy were identified in the TCGA dataset. We included these patients as a control group in our study. Pure GBM patients who received CIK cell immunotherapy in the mITT population showed significantly greater OS rates compared with those of patients in the control group from the TCGA dataset (p = 0.013) **(** **Figure 3A****)**. However, pure GBM patients who received CIK cell immunotherapy in the mITT population showed no significant difference in PFS rates compared with those of patients in the control group from the TCGA dataset **(** **Figure 3B****)**.

#### 3.2.4 Other Predictive Factors Associated With Outcomes

Higher KPS scores and steroid use during the allocated treatment were significantly associated with greater OS rates in multivariate Cox regression analysis for pure GBM patients in the mITT population **(** **Table 2****)**. We also observed that the BMI and degree of surgical resection were significantly associated with greater PFS rates in multivariate Cox regression analysis for pure GBM patients in the mITT population **(** **Table 2****)**.

We have also performed additional subgroup analyses in the mITT population according to age (< 65 years vs ≥ 65 years), sex, patients with non-pure GBM (GBM with astrocytoma, GBM with oligodendroglial tumor, and GBM with others), extent of tumor resection (gross total resection vs subtotal or partial resections or biopsy only), and KPS score (≥ 90 vs < 90) and the results are presented **(** **Supplementary Figures 3–7****)**. We found significant differences in PFS between the CIK immunotherapy and control groups in the ≥ 65 years and gross total resection groups (p = 0.027 and p = 0.009, respectively).

### 3.3 Safety

In the safety population, any grade and ≥ grade 3 adverse events were observed in 84 (98.8%) and 40 (47.1%) patients, respectively, in the CIK cell immunotherapy group and 83 (97.6%) and 31 (36.5%) patients, respectively, in the control group **(** **Table 4****)**. There were no statistically significant differences in the rate of any grade and ≥ grade 3 adverse events **(** **Supplementary Table 2****)**. There were no cases in which treatment had to be discontinued due to treatment-related complications, and other cytokine storm syndromes or anaphylactic reactions were not observed during the clinical trial (18).

**Table 4:** Adverse events of patients in the safety population.

Adverse event	CIK immunotherapy group (n = 85)		Control group (n = 85)	
	All grade	Grade 3 or 4	All grade	Grade 3 or 4
	Number of patients (%)			
Overall incidence	84 (98.8)	40 (47.1)	83 (97.7)	31 (36.5)
Gastrointestinal disorders	73 (85.9)	3 (3.5)	72 (84.7)	3 (3.5)
Skin and subcutaneous tissue disorders	68 (80.0)	1 (1.2)	63 (74.1)	0
Nervous system disorders	69 (81.2)	20 (23.5)	61 (71.8)	16 (18.8)
Metabolism and nutrition disorders	57 (67.1)	6 (7.1)	58 (68.2)	6 (7.1)
Infections and infestations	55 (64.7)	7 (8.2)	46 (54.1)	5 (5.9)
General disorders and administration site conditions	55 (64.7)	5 (5.9)	45 (52.9)	5 (5.9)
Musculoskeletal and connective tissue disorders	40 (47.1)	2 (2.4)	33 (38.8)	0
Investigations	40 (47.1)	11 (12.9)	26 (30.6)	5 (5.9)
Respiratory, thoracic, and mediastinal disorders	31 (36.5)	3 (3.5)	21 (24.7)	2 (2.4)
Eye disorders	20 (23.5)	1 (1.2)	22 (25.9)	0
Blood and lymphatic system disorders	24 (28.2)	10 (11.8)	16 (18.8)	4 (4.7)
Psychiatric disorders	16 (18.8)	0	17 (20.0)	1 (1.2)
Renal and urinary disorders	18 (21.2)	3 (3.5)	12 (14.1)	1 (1.2)
Injury, poisoning, and procedural complications	16 (18.8)	0	7 (8.2)	0
Vascular disorders	9 (10.6)	0	4 (4.7)	0
Ear and labyrinth disorders	7 (8.2)	0	4 (4.7)	0
Reproductive system and breast disorders	6 (7.1)	0	2 (2.4)	0
Immune system disorders	1 (1.2)	0	4 (4.7)	0
Hepatobiliary disorders	0	0	4 (4.7)	1 (1.2)
Cardiac disorders	3 (3.5)	0	1 (1.2)	0
Endocrine disorders	0	0	3 (3.5)	0
Neoplasms: benign, malignant, and unspecified (including cysts and polyps)	0	0	1 (1.2)	0

CIK, cytokine-induced killer.

## 4 Discussion

In a previous phase III randomized trial, we observed that adoptive CIK cell transfer immunotherapy in addition to standard TMZ chemoradiotherapy for patients with GBM led to significantly prolonged PFS but not OS with the maintenance of functional status and quality of life. Serious adverse reactions related to CIK cell immunotherapy were not observed. However, as the study analyzed only the ITT population, the efficacy of CIK cell treatment in GBM patients may not be reflected accurately. The ITT population must include all participants who underwent randomization; thus, some patients had never received immunotherapy and efficacy assessment or deviated from the study criteria. Therefore, we reclassified the ITT population as the mITT population according to the ICH E9 guidelines in this study (27).

In this trial, we observed that adoptive CIK cell immunotherapy in addition to the Stupp protocol (3) may be associated with significant improvements in both OS and PFS after adjusting for other risk factors in patients with pathologically pure GBM. In the Kaplan-Meier curve analysis, there was no significant difference in OS between the CIK cell immunotherapy treatment and control groups (p = 0.079) in patients with pure GBM. However, we observed that the overall degree of tumor resection was relatively lower in the CIK cell immunotherapy group than in the control group in patients with pathologically pure GBM in the mITT population. Gross total resection of GBM substantially improves OS and PFS compared to subtotal resection (28). Therefore, we believe that the difference in the degree of tumor resection is the reason for the lack of significant difference in OS between the CIK cell treatment and control groups in the Kaplan-Meier curve analysis. However, there is a statistically significant difference in OS in the multivariate Cox analysis. When we validated the efficacy of adoptive CIK cell immunotherapy in the independent cohort, OS rates were also improved among pure GBM patients treated with CIK immunotherapy compared with patients from the TCGA dataset. However, there was no significant difference in PFS rates between the CIK immunotherapy group and the control group from the TCGA dataset.

Adoptive cell transfer immunotherapy, which is one of the most encouraging immunotherapies, has shown clinical efficacy and low toxicity in various types of cancers (14). Adoptive cell transfer is a highly personalized cancer therapy that extracts immune cells from patients or donors, amplifies the number of immune cells, and stimulates or modifies antitumor cytotoxicity *ex vivo*, which are then transferred back to the patients (29). CIK cells are the main adoptive immunotherapeutic cells because of their unique biological characteristics and effective therapeutic functions in various cancers (30). Clinical trials related to adoptive CIK cell transfer immunotherapy have been actively conducted worldwide for various cancers, and several trials reported statistically significant improvements in OS and PFS with minimal toxicity (17). CIK cells are known as NK-like T cells with non-MHC-restricted tumor-killing activity and express both the T cell marker CD3 and the NK cell marker CD56 (15). As described in the Introduction section, although the CNS is an immune-privileged site and shows limited immune reactivity, activated T cells can cross the BBB and diffusely expand throughout the brain. Therefore, we believe that CIK cells can cross the BBB and affect GBM cells. To the best of our knowledge, there has been no published clinical trial other than our previous clinical trial on adoptive CIK cell transfer immunotherapy for GBM.

Previously, our study showed that CIK immunotherapy combined with the Stupp regimen improved only PFS but not OS for newly diagnosed GBM patients compared with patients in the control group (18). However, as GBM is known to be occasionally present with anaplastic astrocytoma, oligodendroglioma, or other types of tumors, we sought to examine only patients with pathologically pure GBM (19–22). GBM can induce treatment resistance and an immunosuppressive GBM microenvironment (31–34). In addition, the effect of adjuvant therapy (adjuvant chemotherapy and/or immunotherapy) is associated with the status of the immune microenvironment in GBM (35). Adoptive CIK cell transfer immunotherapy may reverse an immunosuppressive tumor microenvironment and provide a favorable microenvironment that better supports antitumor immunity (36, 37). GBM cancer stem cells can induce an immunosuppressive GBM microenvironment (31–34). Interestingly, it was reported that NK cell cytotoxicity was enhanced against stem cell-like glioblastoma cells compared with differentiated glioblastoma cells (38). In comparison with GBM mixed with other tumors, pure GBM may be more closely associated with an immunosuppressive GBM microenvironment. Therefore, we hypothesized that adoptive CIK cell transfer may show considerable differences in treatment efficacy between the treatment and control groups among patients with pathologically pure GBM without other types of tumors. We reconfirmed the results of the pathology review in this study, which was performed on all participants before randomization, and reclassified the patients according to the pathologic findings for analysis.

In comparison with the control group, the CIK immunotherapy group did not show significant toxicity. In addition, CIK immunotherapy combined with the standard Stupp regimen did not further deteriorate the quality of life (18). The common side effects of CIK cell immunotherapy are mainly grade 1 or 2 toxicities such as fever, chills, fatigue, headache, and skin rash (17). It was reported that grade 3 and 4 adverse events including leukopenia, neutropenia, or thrombocytopenia were considerably rare during CIK cell immunotherapy and tended to be lower in the CIK treatment group compared with the control group (17, 39).

As GBM can induce an immunosuppressive GBM microenvironment, the efficacy of cancer immunotherapy can be reduced (40). GBM cancer stem cells downregulate the expression of MHC molecules to escape cognate antigen recognition by T lymphocytes in an MHC-dependent manner (32). However, as described above, CIK cells are NK-like T cells with non-MHC-restricted tumor-killing activity. Therefore, we believe that CIK cells can effectively overcome immune evasion induced by GBM stem cells. However, TMZ chemoradiotherapy and steroids are associated with the depletion of leukocytes, leading to an immunosuppressive status (41). According to our findings, although corticosteroids have several advantages in GBM treatment, it may be necessary to minimize the administration of steroids during CIK cell immunotherapy for GBM treatment.

A limitation of the study was that there was no information on molecular biomarkers for GBM, including O^6^-methylguanine-DNA methyltransferase (MGMT) gene promoter methylation and isocitrate dehydrogenase 1 (IDH1) gene mutation. In addition, the pathological findings of the tumor tissues of the TCGA cohort are unknown. Therefore, we could not extract patients with pure GBM from the TCGA cohort. Therefore, a simple comparison of the prognosis of patients with pure GBM in the data and patients with not specifically classified GBM in the TCGA data would induce bias in the results. In the future, by reviewing digital pathology images of GBM patients in the TCGA data, we believe that it will be helpful to determine whether adjuvant immunotherapy is associated with improvements in both OS and PFS in patients with pathologically pure GBM from the TCGA data.

In conclusion, this study demonstrated that adjuvant CIK cell immunotherapy in addition to conventional TMZ chemoradiation treatment could prolong OS and PFS in patients with newly diagnosed pathologically pure GBM with no significant adverse events related to treatment. Additionally, compared with patients in the independent cohort, patients treated with CIK cell immunotherapy showed significant improvements in OS rates. We believe that this study may provide guidelines and useful information for investigators who plan to conduct clinical trials involving patients with GBM using adoptive cell transfer immunotherapy. However, this study does not prove the efficacy of adoptive CIK cell therapy for GBM patients. Further studies will be required to validate the findings.

## Data Availability Statement

The original contributions presented in the study are included in the article/**Supplementary Material**. Further inquiries can be directed to the corresponding authors.

## Ethics Statement

The studies involving human participants were reviewed and approved by the local institutional review boards (IRB No. KUH1070007; KMC IRB 0849-01; AN08087; 4-2008-0387; 2008-07-058; 2008-0320) prior to patient enrollment and adhered to the tenets of the Declaration of Helsinki. This trial was also approved by the Ministry of Food and Drug Safety of Korea. The patients/participants provided their written informed consent to participate in this study. Written informed consent was obtained from the individual(s) for the publication of any potentially identifiable images or data included in this article.

## Author Contributions

Conception and design of the study: MH, CK. Analysis of data: MH. Visualization: MH. Manuscript writing: MH, GN. Study supervision: All. Reexamination and revision of the paper: All. All authors read and approved the final manuscript.

## Funding

This study was funded by GC Cell Corp. The funder was not involved in the study design, collection, analysis, interpretation of data, the writing of this article or the decision to submit it for publication.

## Conflict of Interest

Author GN was employed by company GC Cell Corp.

The remaining authors declare that the research was conducted in the absence of any commercial or financial relationships that could be construed as a potential conflict of interest.

## Publisher’s Note

All claims expressed in this article are solely those of the authors and do not necessarily represent those of their affiliated organizations, or those of the publisher, the editors and the reviewers. Any product that may be evaluated in this article, or claim that may be made by its manufacturer, is not guaranteed or endorsed by the publisher.
