# Supplementary material for: Efficacy of Cytokine-Induced Killer Cell Immunotherapy for Patients With Pathologically Pure Glioblastoma
Source: Front Oncol. 2022 Apr 8;12:851628. doi: 10.3389/fonc.2022.851628 (PMC9033287; doi:10.3389/fonc.2022.851628)
Supplement: Supplementary file 1 [file DataSheet_1.docx]

**Supplementary Material**

Efficacy of cytokine-induced killer cell immunotherapy for patients with pathologically pure glioblastoma

Myung-Hoon Han, Jae Min Kim, Jin Hwan Cheong, Je Il Ryu, Yu Deok Won, Gun He Nam, Choong Hyun Kim


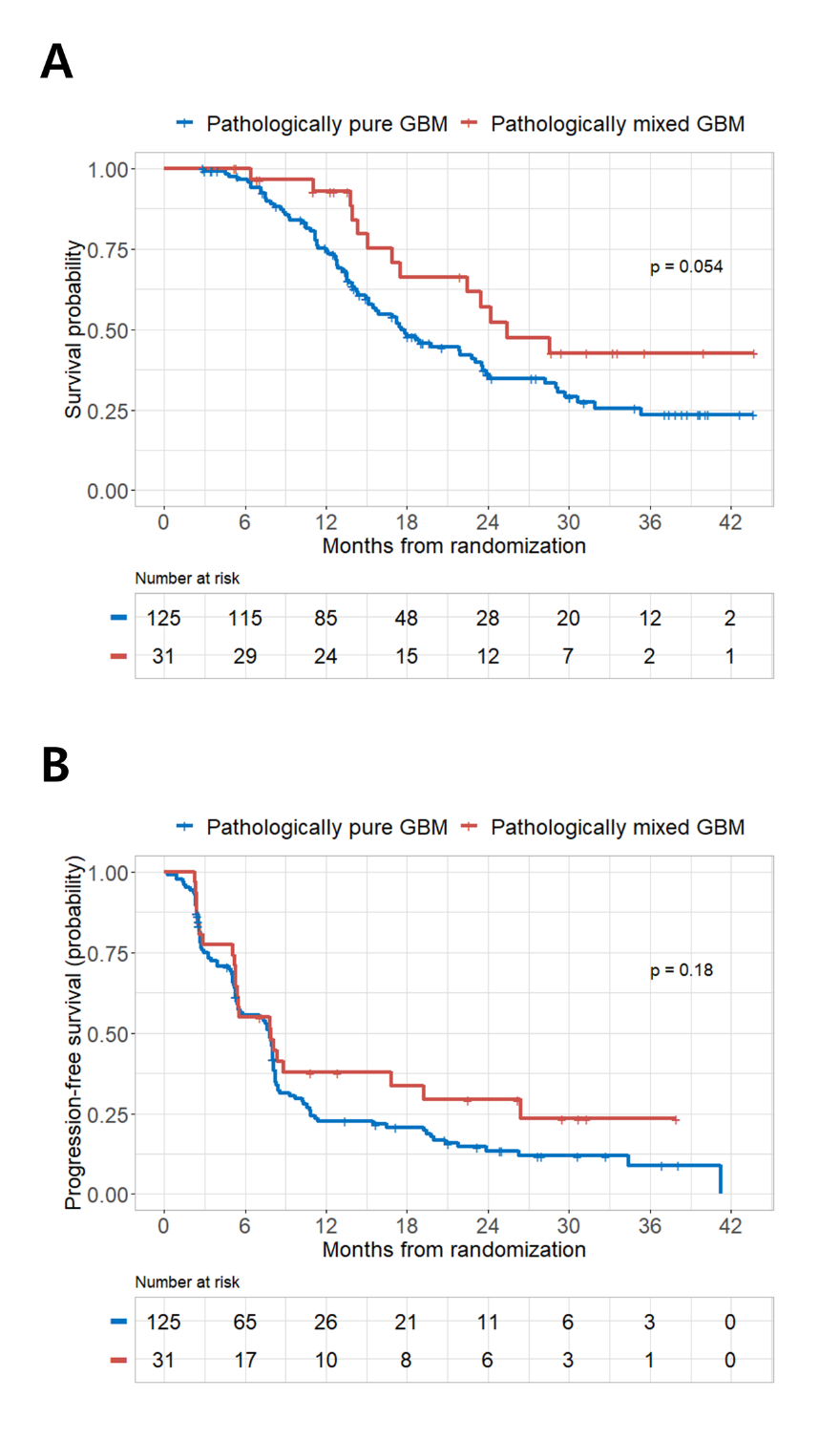


**Supplementary Figure 1.** Kaplan–Meier curves of OS and PFS rates based on the pathologically pure and mixed GBMs in the mITT population. (A) OS rate according to CIK immunotherapy. (B) PFS rate according to CIK immunotherapy.

GBM=glioblastoma multiforme; mITT= modified intention-to-treat; CIK=cytokine-induced killer; OS=overall survival; PFS=progression-free survival.


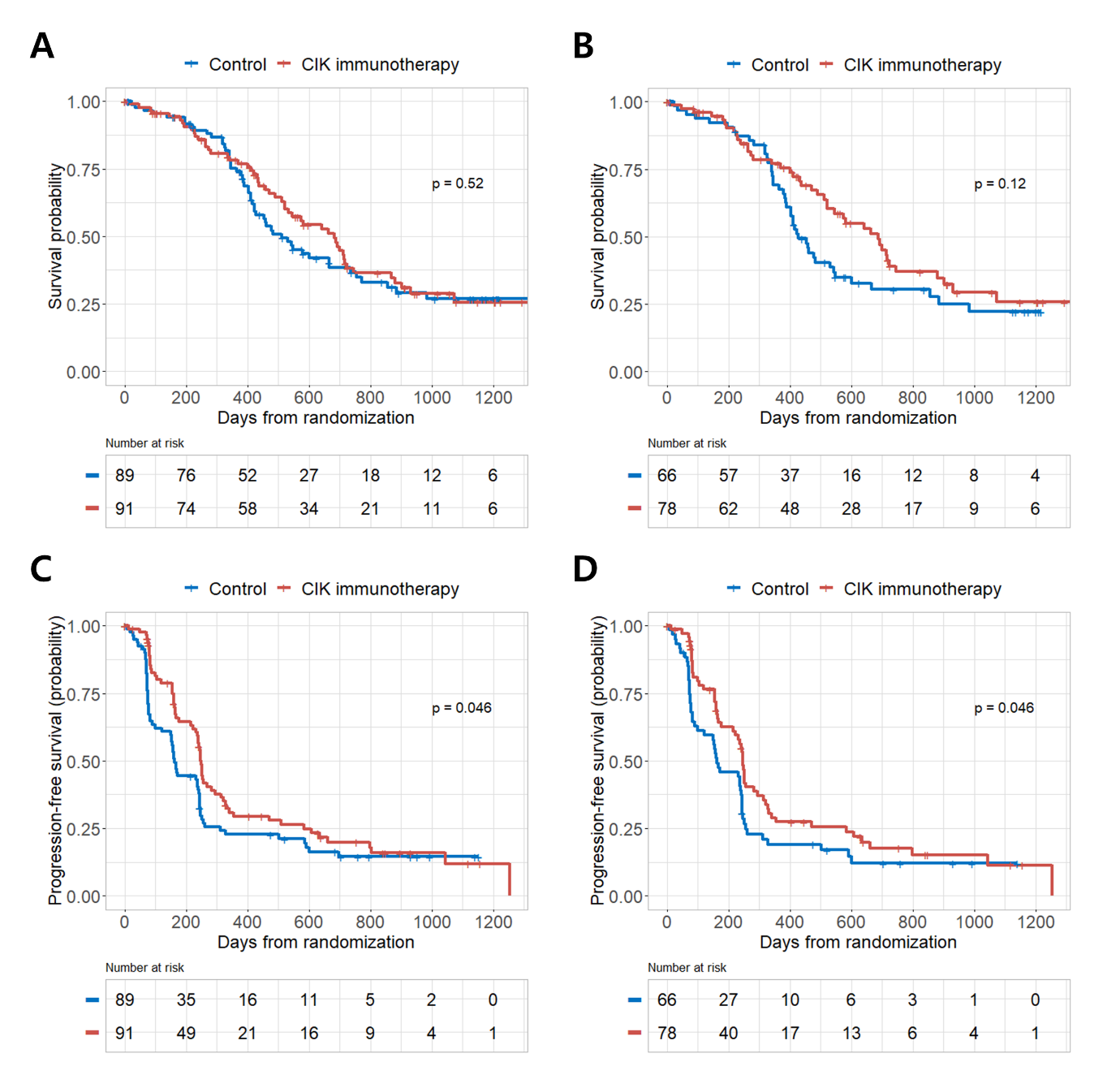


**Supplementary Figure 2.** Kaplan–Meier curves of overall survival (OS) and progression-free survival (PFS) rates according to CIK immunotherapy for the ITT population and patients with pure GBM in the ITT population. (A) OS rate according to CIK immunotherapy for the ITT population. (B) OS rate according to CIK immunotherapy for patients with pure GBM in the ITT population. (C) PFS rate according to CIK immunotherapy for the ITT population. (D) PFS rate according to CIK immunotherapy for patients with pure GBM in the ITT population. CIK=cytokine-induced killer; ITT=intention-to-treat; GBM=glioblastoma multiforme.


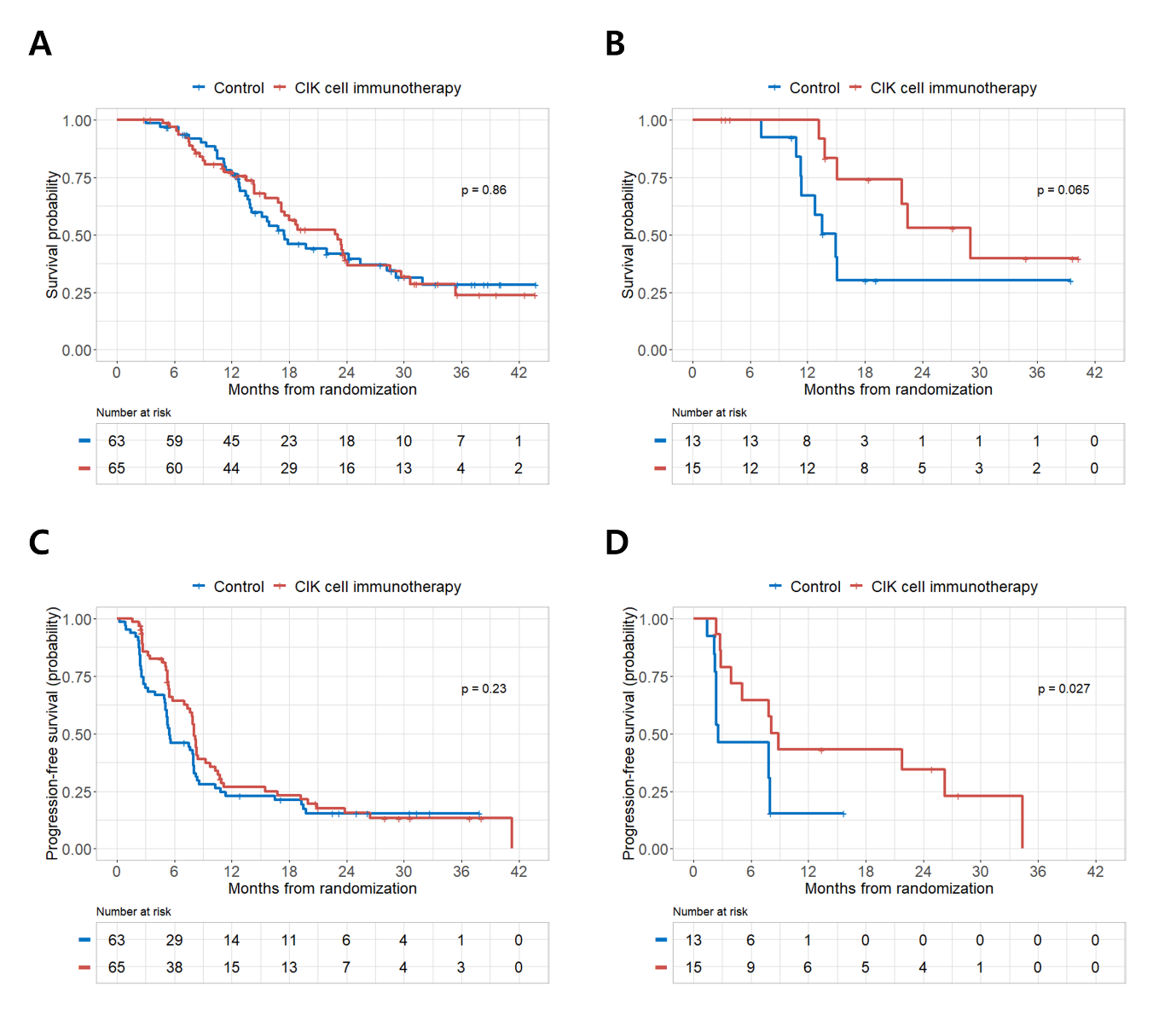


**Supplementary Figure 3.** Kaplan–Meier curves of OS and PFS rates according to CIK immunotherapy based on the age group in the mITT population. (A) OS rate according to CIK immunotherapy in younger patients (<65 years). (B) OS rate according to CIK immunotherapy in older patients (≥65 years). (C) PFS rate according to CIK immunotherapy in younger patients (<65 years). (D) PFS rate according to CIK immunotherapy in older patients (≥65 years).

CIK=cytokine-induced killer; mITT= modified intention-to-treat; OS=overall survival; PFS=progression-free survival.


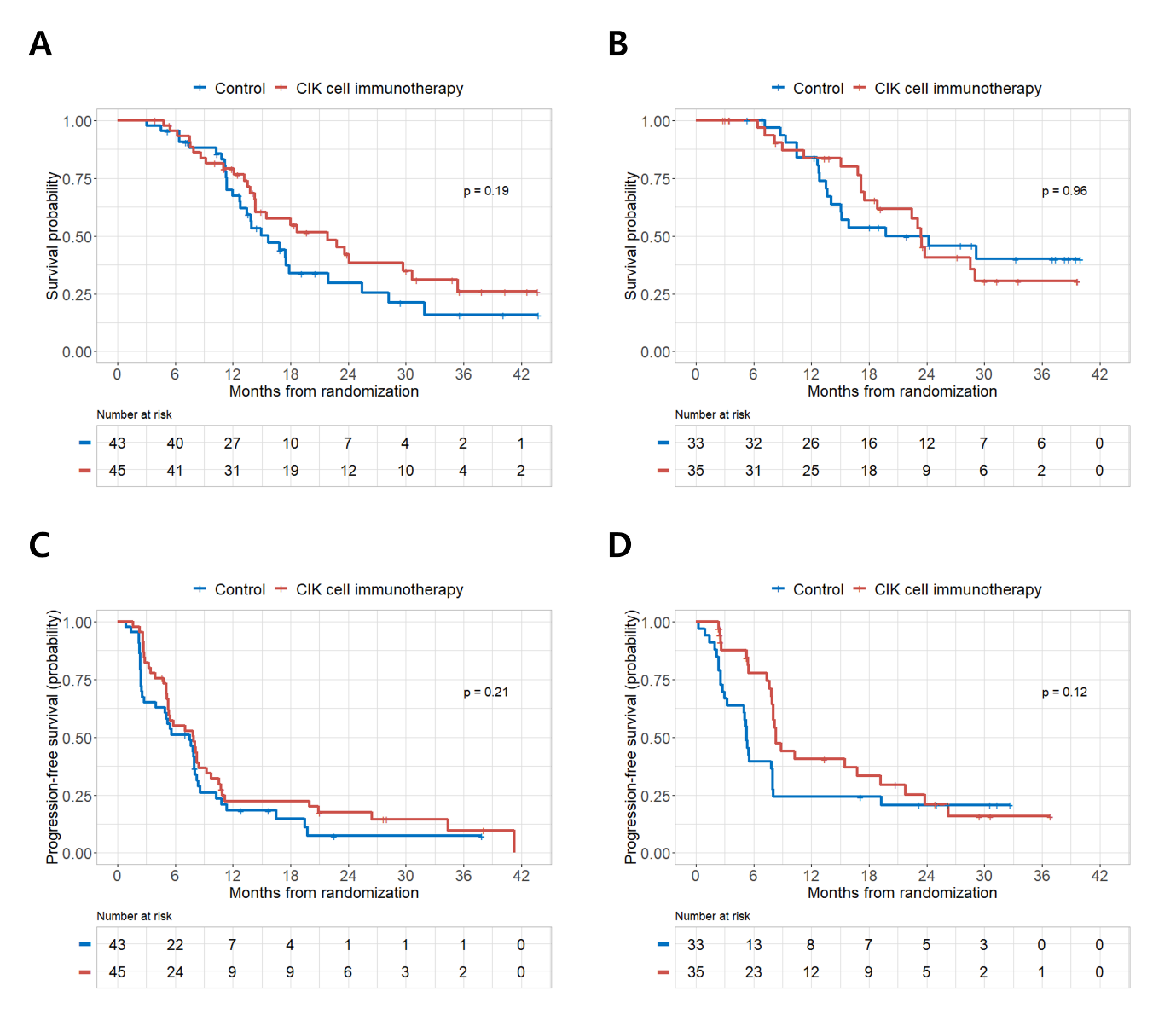


**Supplementary Figure 4.** Kaplan–Meier curves of OS and PFS rates according to CIK immunotherapy based on the sex in the mITT. (A) OS rate according to CIK immunotherapy in male patients. (B) OS rate according to CIK immunotherapy in female patients. (C) PFS rate according to CIK immunotherapy in male patients. (D) PFS rate according to CIK immunotherapy in female patients.

CIK=cytokine-induced killer; mITT=modified intention-to-treat; OS=overall survival; PFS=progression-free survival.


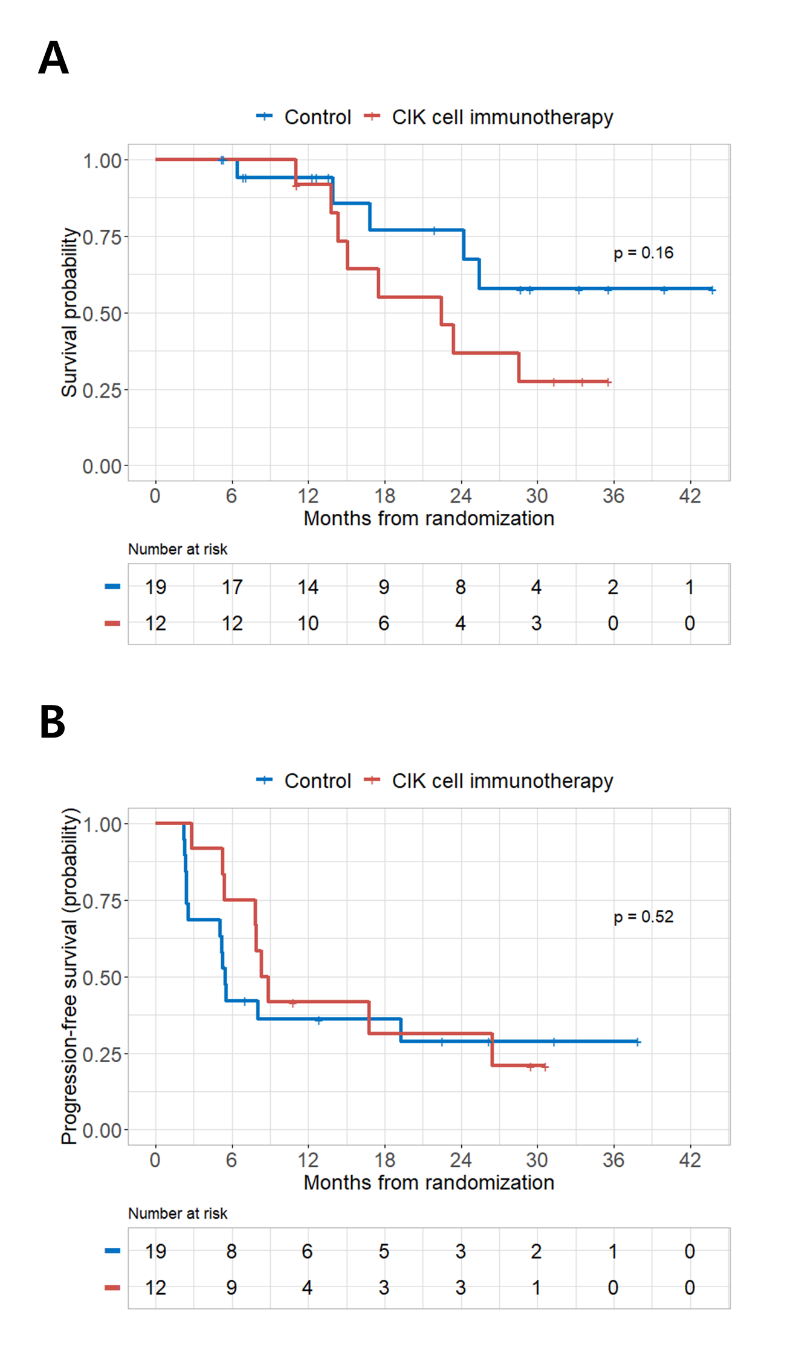


**Supplementary Figure 5.** Kaplan–Meier curves of OS and PFS rates according to CIK immunotherapy in patients with pathologically mixed GBMs (GBM with astrocytoma, GBM with oligodendroglial tumor, and GBM with others) in the ITT population. (A) OS rate according to CIK immunotherapy. (B) PFS rate according to CIK immunotherapy.

CIK=cytokine-induced killer; GBM=glioblastoma multiforme; mITT=modified intention-to-treat; OS=overall survival; PFS=progression-free survival.


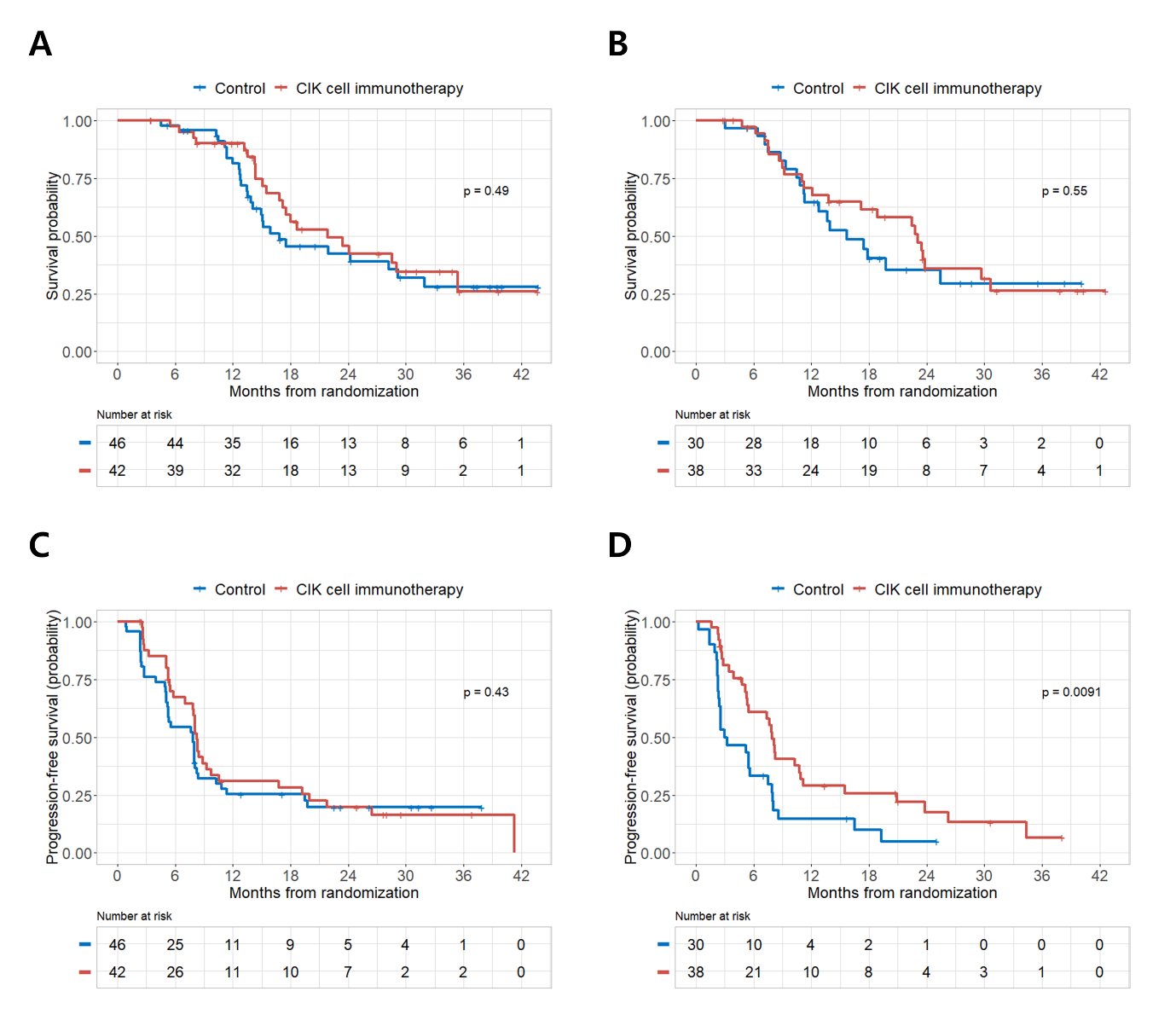


**Supplementary Figure 6.** Kaplan–Meier curves of OS and PFS rates according to CIK immunotherapy based on the extent of tumor resection in the mITT population. (A) OS rate according to CIK immunotherapy in gross total resection group. (B) OS rate according to CIK immunotherapy in less than gross total resection group. (C) PFS rate according to CIK immunotherapy in gross total resection group. (D) PFS rate according to CIK immunotherapy in less than gross total resection group.

CIK=cytokine-induced killer; mITT=modified intention-to-treat; OS=overall survival; PFS=progression-free survival.


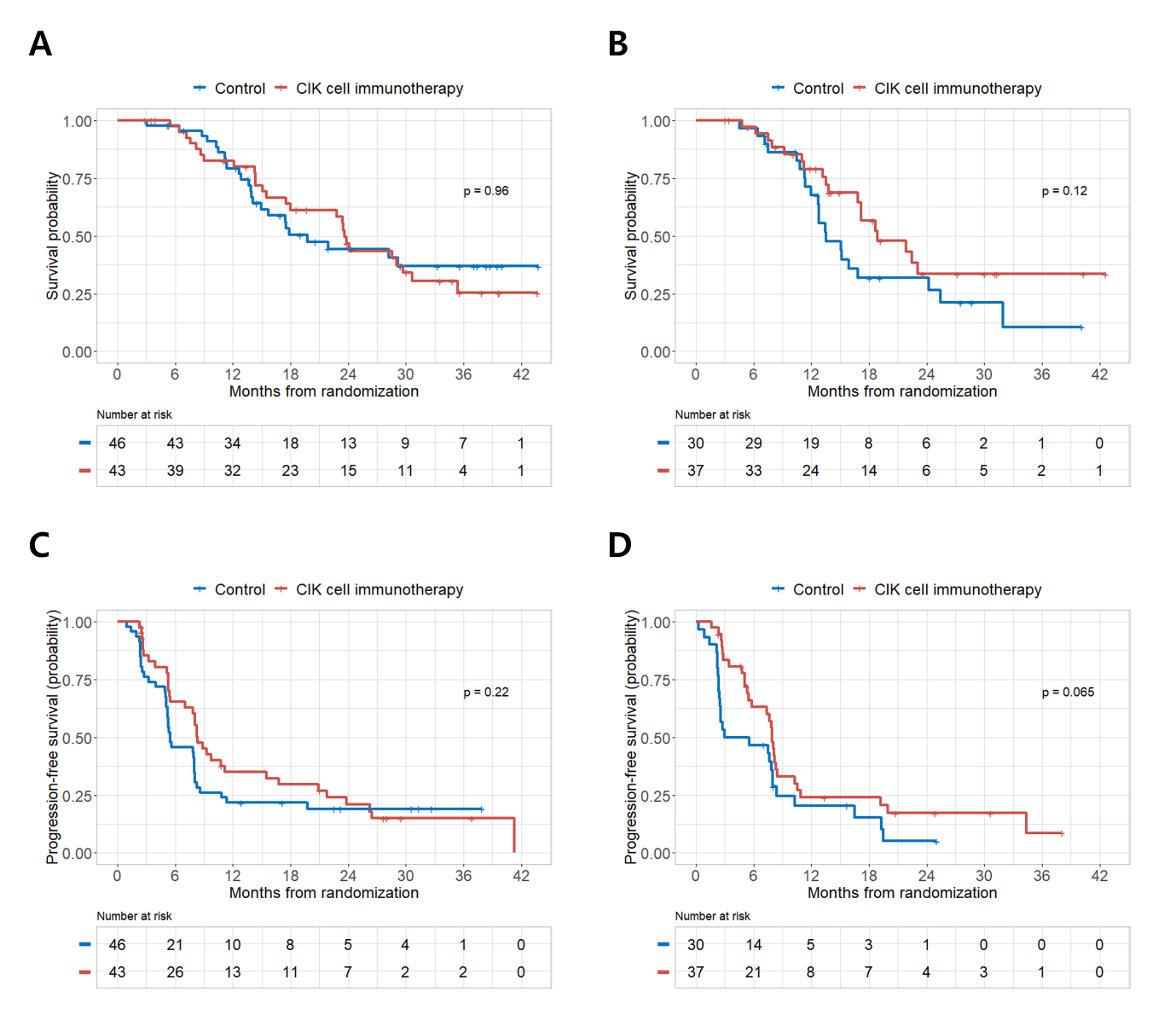


**Supplementary Figure 7.** Kaplan–Meier curves of OS and PFS rates according to CIK immunotherapy based on the KPS score in the mITT population. (A) OS rate according to CIK immunotherapy in patients with higher KPS score (≥ 90). (B) OS rate according to CIK immunotherapy in patients with lower KPS score (< 90). (C) PFS rate according to CIK immunotherapy in patients with higher KPS score (≥ 90). (D) PFS rate according to CIK immunotherapy in patients with lower KPS score (< 90).

CIK=cytokine-induced killer; KPS= Karnofsky performance status; mITT=intention-to-treat; OS=overall survival; PFS=progression-free survival.

**Supplementary Table 1.** Overall survival and progression-free survival analyses according to the clinical parameters of patients with pure GBM in the ITT population

|  | Overall survival | | | |  | Progression-free survival | | | |
| --- | --- | --- | --- | --- | --- | --- | --- | --- | --- |
|  | Univariate analysis | | Multivariate analysis | |  | Univariate analysis | | Multivariate analysis | |
| Variable | HR (95% CI) | p | HR (95% CI) | p |  | HR (95% CI) | p | HR (95% CI) | p |
| Sex |  |  |  |  |  |  |  |  |  |
| Male | Reference |  | Reference |  |  | Reference |  | Reference |  |
| Female | 0.67 (0.43–1.04) | 0.076 | 0.59 (0.38–0.92) | **0.019** |  | 0.87 (0.59–1.29) | 0.486 | 0.80 (0.54–1.20) | 0.288 |
| Age (per 1 year increase) | 1.01 (0.99–1.03) | 0.303 | 1.00 (0.98–1.02) | 0.950 |  | 1.00 (0.98–1.02) | 0.981 | 1.00 (0.98–1.02) | 0.858 |
| Karnofsky performance scale score  (per 10 score decrease) | 1.16 (0.99–1.37) | 0.073 | 1.24 (1.03–1.50) | **0.023** |  | 1.04 (0.90–1.21) | 0.572 | 1.09 (0.92–1.29) | 0.326 |
| Extent of resection |  |  |  |  |  |  |  |  |  |
| Biopsy only | Reference |  | Reference |  |  | Reference |  | Reference |  |
| Subtotal or partial resection | 1.19 (0.53–2.67) | 0.676 | 1.31 (0.56–3.04) | 0.531 |  | 0.40 (0.20–0.82) | **0.012** | 0.37 (0.18–0.78) | **0.009** |
| Gross total resection | 0.88 (0.39–1.95) | 0.745 | 0.84 (0.37–1.92) | 0.677 |  | 0.39 (0.20–0.78) | **0.007** | 0.30 (0.14–0.64) | **0.002** |
| Steroid use |  |  |  |  |  |  |  |  |  |
| No | Reference |  | Reference |  |  | Reference |  | Reference |  |
| Before allocated treatment | 0.79 (0.11–5.94) | 0.822 | 0.63 (0.08–4.76) | 0.652 |  | 0.40 (0.05–2.98) | 0.373 | 0.36 (0.05–2.70) | 0.321 |
| During allocated treatment | 2.03 (1.16–3.54) | **0.013** | 1.94 (1.10–3.43) | **0.023** |  | 1.72 (1.04–1.81) | **0.036** | 1.59 (0.94–2.70) | 0.087 |
| Both before and during allocated treatment | 1.28 (0.72–2.29) | 0.401 | 1.01 (0.54–1.87) | 0.985 |  | 1.09 (0.66–1.83) | 0.732 | 0.89 (0.51–1.55) | 0.673 |
| Treatment group |  |  |  |  |  |  |  |  |  |
| Control group | Reference |  | Reference |  |  | Reference |  | Reference |  |
| CIK immunotherapy group | 0.72 (0.47–1.10) | 0.124 | 0.62 (0.39–0.97) | **0.036** |  | 0.68 (0.46–1.00) | **0.049** | 0.58 (0.38–0.88) | **0.011** |
| GBM, glioblastoma multiforme; ITT, intention-to-treat; HR, hazard ratio; CI, confidence interval; CIK, cytokine-induced killer; p < 0.05 is shown in bold. | | | | | | | | | |

**Supplementary Table 2.** Adverse events in the safety population

| Type | CIK immunotherapy group (n = 85) | | Control group  (n = 85) | | p |
| --- | --- | --- | --- | --- | --- |
|  | n (%) | Total cases | n (%) | Total cases |  |
| Adverse event | 84 (98.8) | 1416 | 83 (97.7) | 1061 | 1.000 |
| Treatment-emergent adverse event | 84 (98.8) | 1334 | 83 (97.7) | 1020 | 1.000 |
| Adverse drug reaction* | 16 (18.8) | 57 | – | – |  |
| Grade 3/4 adverse event | 40 (47.1) | 114 | 31 (36.5) | 64 | 0.162 |

*****Associated with a causational relationship between the investigational product and the event.

CIK, cytokine-induced killer.
